# Molecular tags for electron cryo-tomography

**DOI:** 10.1042/ETLS20240006

**Published:** 2025-12-23

**Authors:** Emma Silvester, Lindsay A. Baker

**Affiliations:** 1 Department of Biochemistry, University of Oxford, Oxford OX1 3QU, U.K; 2 Kavli Institute for Nanoscience Discovery, University of Oxford, Dorothy Crowfoot Hodgkin Building, South Parks Road, Oxford OX1 3QU, U.K

## Abstract

Electron cryotomography enables the direct visualisation of biological specimens without stains or fixation, revealing complex molecular landscapes at high resolution. However, identifying specific proteins within these crowded environments is challenging. Molecular tagging offers a promising solution by attaching visually distinctive markers to proteins of interest, differentiating them from the background. This review explores available tagging strategies, including gold nanoparticles, metal-binding proteins, nucleic acid nanostructures and protein-based tags. The identification and targeting strategies for each approach are discussed, highlighting their respective advantages and limitations. Future directions for advancing these tagging techniques to expand their applicability to broader research questions are also considered.

## Introduction

Cellular functions are rarely driven by single molecules acting in isolation; instead, they emerge from the complex interplay between many molecular species. To fully understand cellular processes, it is essential to study not only the structure of biomolecules, but also their interactions with the surrounding cellular milieu. Advances in super-resolution fluorescence microscopy (FM) enable us to probe cells at the molecular level. However, since only labelled molecules are detected, much of the cell remains unseen. Cryogenic electron tomography (cryoET) provides a more complete view, by imaging cells or other heterogeneous biological samples like viruses and vesicles in a native hydrated state without stains or chemical fixation. As cryoET captures all the molecules present simultaneously, interpreting this data presents challenges, including crowding and low signal-to-noise ratios (SNR). To overcome these challenges, tagging methods to identify specific molecules of interest are needed.

### Tagging strategies

While cryoET has the potential to image the entire molecular contents of a cell, parsing this data is challenging. Biological specimens, composed mainly of light atoms, scatter electrons weakly. This characteristic, combined with their radiation sensitivity, results in a low SNR [1]. Additionally, the crowded cellular environment complicates the identification specific molecules, especially without prior structural information.

There are two routes for protein identification in cryoET: indirect localisation using correlative light electron microscopy (CLEM) and direct identification by attaching a high contrast ‘molecular tag’ to the protein of interest, which can be unambiguously identified in tomograms.

### CLEM

CLEM integrates light microscopy, usually FM, with electron microscopy (EM) to provide complementary information. A key advantage is the use of established genetic fusion tags to generate the fluorescence signal, meaning that all target molecules are labelled. The FM is typically performed under cryogenic conditions, which presents technical challenges. In diffraction-limited cryoFM, the achievable resolution is limited to ~400 nm in the *xy*-plane and ~1 μmm in the *z*-plane [2,3]. Super-resolution cryoFM can theoretically achieve sub-nanometre localisation precision, but in practice, the high photon flux required can cause specimen melting or damage, limiting the achievable resolution to ~50–100 nm [4,5].

Due to these resolution limits, CLEM is usually used for guiding data acquisition (e.g. identifying specific cells expressing the fluorescent target protein) or locating the general region of a tomographic volume containing the fluorescent signal (e.g. a particular organelle), rather than pinpointing the molecules corresponding to specific densities in a tomogram [3].

### Molecular tags

**Figure 1:**
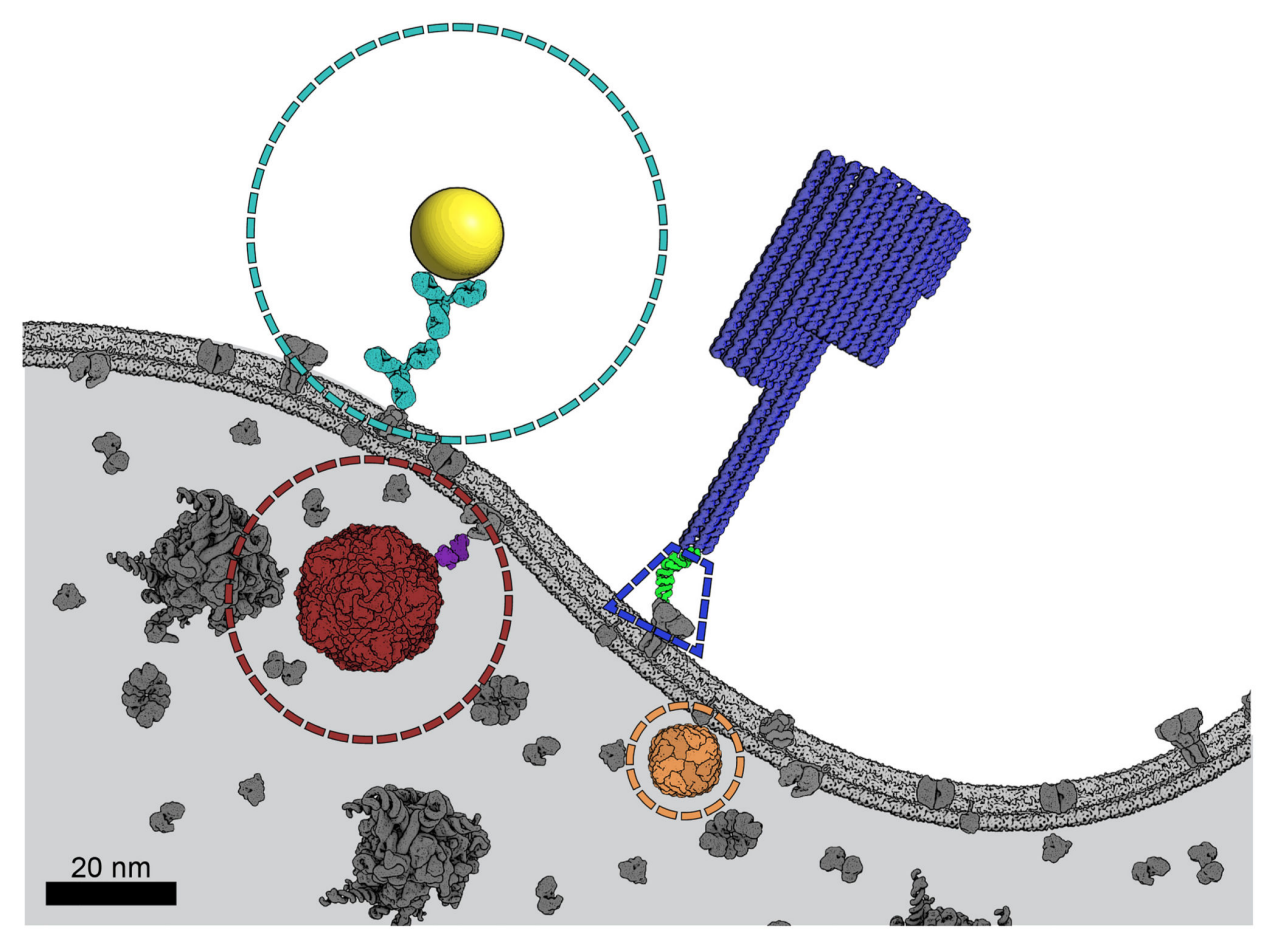
Principles for molecular tagging in cryoET. Molecular tags must contain an easily identifiable component such as gold nanoparticles (yellow) [6–9], metal-binding proteins such as ferritin (orange) with its iron core [10–15], DNA nanostructures (blue) [16], or distinctive protein assemblies like encapsulin (red) [17]. Additionally, molecular tags require a component that binds to the protein of interest, such as an antibody (cyan) [7,18], an aptamer (green) [16], a targeting polypeptide like the FKBP/FRB dimerisation system [11,17], or a direct genetic fusion as show with ferritin [10,12–15]. Generally, the targeting component may not be visible, and the protein of interest may exist anywhere within a region of possible locations. This region depends on the size and valency/symmetry of the tag, as well as the size of the targeting component. Illustrative regions of possible locations for these examples are indicated with dashed lines.

The second approach involves attaching a ‘molecular tag’ to the target protein. These tags feature a high-contrast component for visibility in tomograms and a targeting component for binding to the molecule of interest (Figure 1).

The high-contrast component typically incorporates clusters of strongly scattering elements like gold, iron, or phosphorous, although protein-only tags are also used. Targeting strategies include genetic fusion or post-translational targeting through additional elements such as antibodies or aptamers.

Most molecular tags are symmetrical, meaning the target protein may exist anywhere within a radius around the tag (Figure 1). The radius depends on the size of the tag components and defines the targeting precision. Asymmetric tags with a precisely positioned targeting component enable more accurate localisation of the target.

### Strategies for identification

To identify molecular tags in cryoET, an easily identifiable component must be incorporated. Current strategies include synthetic gold nanoparticles, metal-binding proteins, nucleic acid nanostructures, and large, symmetric protein-only tags.

#### Gold nanoparticles

**Figure 2:**
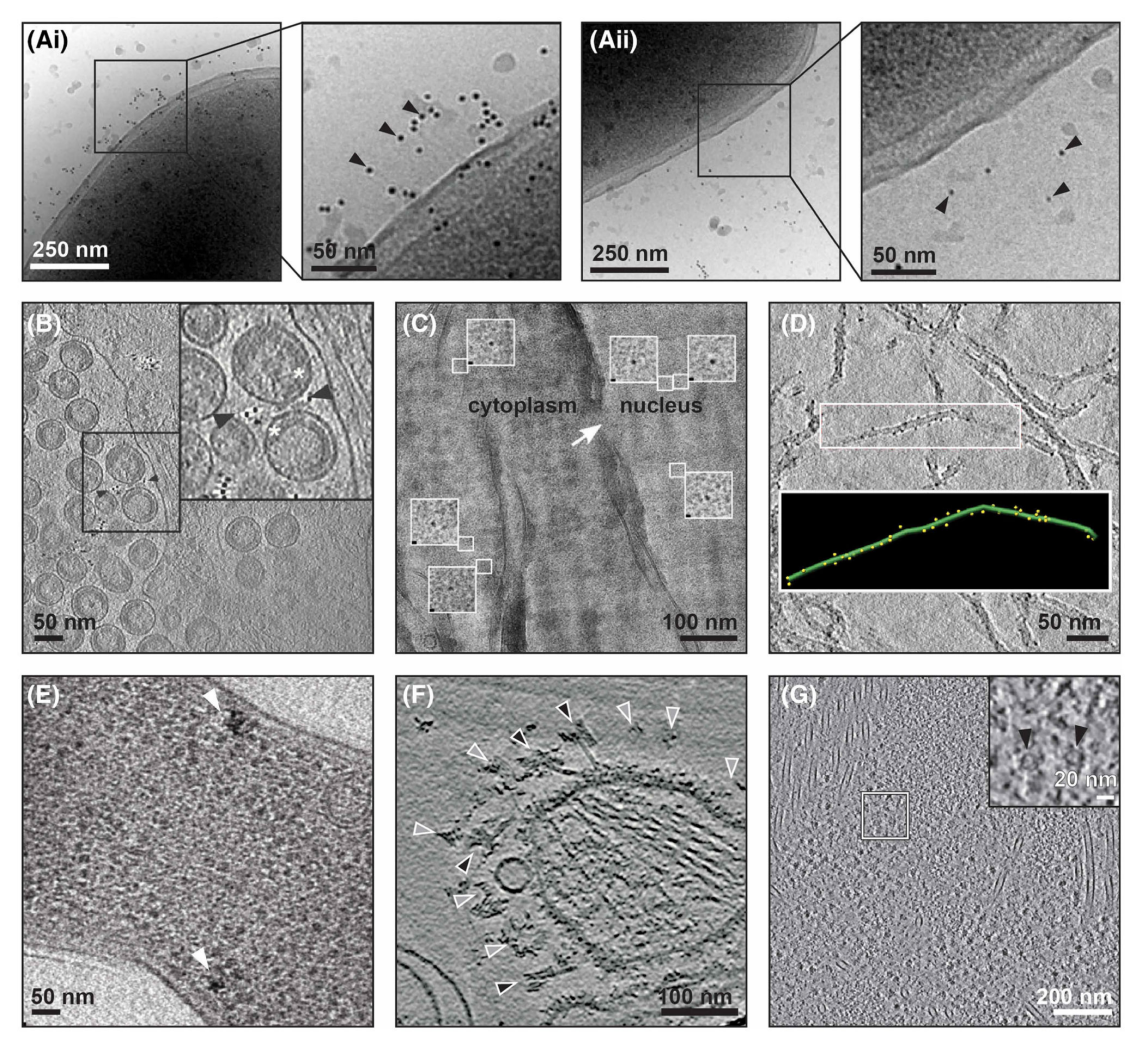
Examples of molecular tags for cryoET. **(A)** Nanobody-conjugated gold nanoparticles (black arrowheads) label the surface protein CdrA on *P. aeruginosa* cells in cryoEM projection images: with (i) and without (ii) CdrAB overexpression. Adapted from Melia et al. [8]. **(B)** Immunogold-labelled tetherin (black arrowheads) is visualised on the surface of HIV virus-like particles attached to HT1080 cells in a computed tomographic slice (slice thickness 7.64 nm). Adapted from Yi et al. [7]. **(C)** Electroporated PEG-coated 2 nm gold nanoparticles, surface-functionalised with SV40 nuclear localisation signal (NLS) containing peptides, are visualised near the nuclear envelope of sectioned HeLa cells in cryoEM projection images. The white arrow indicates a nuclear pore. Adapted from Groysbeck et al. [9]. **(D)** Tomographic slice of desmin-metallothionein intermediate filaments after incubation in Au(I)Cl, with gold clusters visible along the filament surface. The inset shows a surface-rendered representation of the area marked by the red box. Adapted from Bouchet-Marquis et al. [13]. **(E)** Ferritin (white arrowheads) marks the position of the protein ZapA in a computed tomogram slice during cell division in *E coli* (slice thickness 10 nm). Adapted from Wang et al. [10]. **(F)** DNA origami-based tags (SPOTs) bind to membrane vesicles containing the glycoprotein B from herpes simplex virus I in a computed tomographic slice (slice thickness 2.8 nm). SPOTs with their identification post at least partially in plane with the slice are indicated with black and white arrowheads; those with their posts out of plane are shown with grey and white arrowheads. Adapted from Silvester et al. [16]. **(G)** Localisation of encapsulin-based tags (GEMs) on the EGFP-Ki-7-coated mitotic chromosome periphery as seen in a computed tomographic slice. Example GEM particles are indicated with black arrowheads in the inset. Adapted from Fung et al. [17].

The classical immunogold technique, in which nanoparticles are conjugated to secondary antibodies, was introduced in 1971 [19], and has since become a staple of room-temperature EM. Intracellular labelling typically requires chemical fixation and permeabilisation, but native immunogold labelling has been demonstrated for isolated nuclei [6], viral glycoproteins (Figure 2B) [7], cell surface proteins [7], and isolated membranes [18]. In addition to conventional workflows using primary and secondary antibodies, single-chain antibodies (nano-bodies) and antibody derivatives can be used. For example, nanobody-conjugated gold nanoparticles were recently used to locate CdrA on *Pseudomonas aeruginosa* outer membrane protrusions (Figure 2A).

The strong scattering of metallic nanoparticles provides high contrast but also causes artefacts during tomogram reconstruction and can obscure biological features [20]. Using smaller gold clusters helps mitigate these artefacts and reduces the non-specific binding that is prevalent with colloidal gold [9,21,22].

Recently, polyethylene glycol (PEG)-coated 2 nm gold nanoparticles were proposed as intracellular molecular tags [9]. The PEG coating minimises clustering and non-specific interactions. Although these nanoparticles, provide lower contrast than larger colloidal particles, they are still detectable in cryoEM micrographs (Figure 2C). While specific targeting inside cells has not yet been demonstrated, this approach shows promise for future developments.

#### Metal binding proteins

Alternatively to synthetic nanoparticles, metal clusters can be introduced via metal-binding protein genetic fusion tags such as metallothionein. Metallothionein sequesters metal ions, forming electron-dense clusters when exposed to metal salts. Proposed as EM tags in 2007 [12], metallothionein tags have been used to identify proteins in freeze-substituted *Escherichia coli* cell sections and on native isolated filaments (Figure 2D) [13,15]. However, their application in cryoET has been limited by technical challenges. The tags provide relatively low contrast due to the small number of metal-binding sites present (7–12 per metallothionein), often necessitating multiple copies of the tag for visualisation [23,24]. Additionally, incorporating metallothionein fusions into protein complexes is challenging and can cause artificial oligomerisation of the target protein [12]. Overexpression of metallothionein can impede cell growth, and the high metal concentrations required for the tag to become metal-bound can be toxic to cells and can induce native metallothionein expression, complicating data analysis [13,15].

To address some of these concerns, bacterial ferritin was proposed as an alternative (Figure 2E) [10]. Ferritin uses multiple subunits to create large iron clusters, typically ~2000 atoms [25], providing much higher contrast. Growing cells in iron-rich media is less toxic than the heavy metals required for metallothionein labelling, although may not be viable for all eukaryotic cell lines [11]. The Ferritag system, which uses human ferritin particles, was developed to overcome technical issues with the original bacterioferritin method and to extend applicability to mammalian cells [11]. Ferritag particles are not genetically fused to the target protein, as fusion can result in mis-localisation and aggregation [10,11]. Instead, they are expressed independently and inducibly targeted to the protein of interest. Currently, iron-loaded Ferritag labelling has only been demonstrated with fixed, resin-embedded cells, but the method should translate to cryoET since the iron clusters form in the cell prior to fixation.

Ferritin is a naturally occurring ‘cloneable nanoparticle’. Other similar cloneable nanoparticles can be engineered as well. For example, expressing encapsulin subunits alongside ferritin-like cargo results in larger iron cores [26,27]. Encapsulin shells of different sizes can be generated, enabling potential multiplexed labelling [28]. A comparable approach uses genetic fusions of metal reductases and metal-binding peptides to produce nanoparticles of controlled sizes, whilst also conferring resistance to otherwise toxic metal salts [29]. This method has successfully produced cloneable Selenium nanoparticles [29], Tellurium nanoparticles [30], and Cadmium Selenide quantum dots [31]. These systems have been proposed as contrast agents for cryoET, although specific protein targeting or expression in eukaryotic cells remains to be demonstrated.

#### Nucleic acid nanostructures

Nucleic acids provide an identification strategy which does not rely on heavy metals. Phosphorus elastically scatters ~4× more electrons than carbon, oxygen or nitrogen [32], making nucleic acids some of the highest contrast elements in cellular tomograms. The DNA origami method facilitates the precise assembly of large 3D nanostructures *in vitro* which are easily identifiable among other biomolecules in cryoET [33–36]. DNA origami ‘signposts’ (SPOTs) have been used to identify individual protein molecules on vesicles, viruses, and the cell surface, although intracellular labelling has not yet been demonstrated (Figure 2F) [16]. In the context of surface labelling, these nanostructures are also identifiable at low magnification, making them useful for selecting areas for data acquisition.

Another promising direction is the use of single-strand RNA nanostructures. RNA sequences can be designed to fold co-transcriptionally into well-defined 3D shapes, making use of tertiary contacts between single-stranded regions in the RNA secondary structure [37,38]. Such nanostructures have been shown to fold in *E. coli* [39–41], suggesting the potential for a genetically encoded tag. Although most reported RNA nanostructures are too small be effective cryoET tags, new computational design tools have led to the design of 3D nanostructures over 2 kb in size [42]. Larger nanostructures typically have a decreased yield of correctly folded products, but new insights into RNA folding from cryoEM reconstructions of RNA nanostructures could lead to higher fidelity designs suitable for cryoET [43].

#### Protein-based tags

Recently, protein-only tags have also been proposed for cryoET. Genetically encoded multimeric particles (GEMs) are icosahedral protein shells that assemble from modified encapsulin sub-units [17]. Although these tags lack heavy elements and are thus lower in contrast than previously described tags, they are identifiable in reconstructed tomograms due to their large size (~25 nm) and distinctive shape (Figure 2G).

In a similar vein, an iron-free adaptation of the FerriTag system has been recently introduced [44]. By omitting iron loading, this strategy minimises contrast artifacts and eliminates the requirement for iron-rich growth conditions. Iron-free FerriTag particles were used to locate and visualise clathrin-associated proteins in ‘unroofed’ cells, although it is unclear if these tags would provide sufficient contrast for tagging in thicker samples such as cell sections.

Protein origami presents another potential avenue for cryoET tags. This method uses orthogonal coiled-coil interactions to generate polypeptide polyhedra from one or more chains [45–47]. Although currently reported structures are too small to serve as cryoET tags, adding more polypeptide domains or careful incorporation of proteins at polyhedra vertices could potentially yield suitable tags.

### Strategies for targeting

Molecular tags must not only feature a high-contrast component for identification but must also co-localise with their target. This may be accomplished via direct genetic fusion or by incorporating molecules which bind the target protein. Targeting molecules can be polypeptides, like antibodies, or non-polypeptides, such as nucleic acids or small molecules.

Key considerations in selecting a targeting strategy include: coverage (fraction of target proteins tagged), specificity (fraction of tags bound to target proteins), targeting precision (the smallest spatial region to which the target can be localised), and biological impact (effect on target protein folding and localisation). The relative importance of these factors will depend on the biological question being addressed.

Molecular tags may incorporate one or more copies of the targeting component, resulting in monovalent or multivalent labelling, respectively. This distinction affects both the precision and biological impact of targeting.

#### Genetic fusions vs. post-translational targeting

The most direct method to target a specific protein is through a genetic fusion, where the molecular tag is incorporated into the gene of interest. This ensures optimal specificity and coverage since, theoretically, all target proteins possess the tag, and the tag should not be expressed elsewhere in the cell. Currently, only metal-binding proteins like metallothionein and ferritin have been used as genetic fusion tags for cryoET [10,13]. However these methods face technical issues: cells must be grown with high concentrations of metal salts, the low metal-binding capacity of monomeric metallothionein fusions results in a poor SNR [13], and the multimeric nature of ferritin fusions can cause mis-localisation and aggregation of the target [10,11].

Due to these limitations, most cryoET tags are synthesised or expressed independently of the protein of interest and targeted post-translationally. This approach results in some target proteins being untagged and a population of unbound/background tags, i.e. reduced coverage and specificity. However, post-translational targeting is more adaptable: there are no specific buffer requirements, and targeting strategies can be optimised to minimise biological impact.

A common, modular approach for post-translational targeting involves directing the tag towards a genetic fusion on the protein of interest, such as a fluorescent protein [16,17]. This approach allows for versatile use of the molecular tag across different proteins with the same fusion, reducing the need to develop new tags. Target protein constructs with common fusions like GFP are often readily available and well-characterised. For cases where genetic fusions are not viable, tags which target the native protein can be used instead. This can be achieved using, for example, a nanobody.

#### Polypeptide-based vs. non-polypeptide-based targeting

For genetically encoded post-translational tags such as GEMs and Ferritags, the targeting component must be a polypeptide that binds the protein of interest, such as a nanobody or a receptor. Exogenous tags, such as gold nanoparticles and SPOTs, can also incorporate polypeptide-based targeting components. Several established methods exist for functionalising DNA nanostructures and gold nanoparticles with proteins [48].

A pertinent example of polypeptide-based targeting is the FKBP-rapamycin-FRB heterodimerisation used by Ferritags [11]. Here, proteins of interest possess a FKBP fusion, while the Ferritag incorporates an FRB fusion. Binding is induced by rapamycin. GEM tags also use this strategy, but an additional adaptor molecule (a GFP nanobody-FKBP fusion) is introduced [17,49]. This addition increases the overall size and complexity of the tag but allows targeting of commonly used GFP fusion proteins. Additionally, the GFP nanobody could be swapped out for other nanobodies, enabling targeting to native proteins.

Exogenous tags like SPOTs and gold nanoparticles can alternatively incorporate non-polypeptide-based targeting elements, such as aptamers and small molecules. Aptamers can be selected to bind a wide range of targets and are generally less costly to develop than nanobodies [50]. Small molecule ligands, such as benzyl-guanine which binds SNAP tags, offer the advantage of covalent binding, potentially improving coverage and specificity over non-covalent targeting strategies. Oligonucleotides functionalised with these small molecules are commercially available and could easily be added to existing SPOTs or gold nanoparticle tags.

#### Monovalent vs. multivalent labelling

One important consideration when choosing a targeting strategy is monovalent versus multivalent labelling. Most molecular tags, including nanoparticles, Ferritags and GEMs, contain multiple copies of the targeting component. Consequently, these tags can bind multiple target proteins, leading to clustering or aggregation. This issue is especially pronounced for the original bacterioferritin tag implementation in which the ferritin subunit is genetically fused to the target protein [10,11]. The rapamycin-induced FKBP-FRB heterodimerisation mechanism used by GEMs and Ferritags helps reduce clustering effects by providing temporal control over binding [11,17].

Multivalent tags also limit targeting precision as the target protein may exist anywhere within a region around the tag (Figure 1). In crowded environments without a clear reference structure, such as a membrane, this limitation hampers our ability to identify the exact molecule being targeted. Currently, SPOTs are the only established molecular tags that are both asymmetric and monovalent, although future RNA or protein origami tags might also fulfil these criteria.

#### Tags for sub-volume averaging

While this review focuses on molecular tags which can be identified in single tomograms, there is another class of tags which require sub-volume averaging to reveal their location. Sub-volume averaging, which involves extracting and averaging sub-tomograms of repeating structures, can be used to generate high resolution reconstructions of macromolecular complexes. Tags can be incorporated into constituent proteins within these complexes. Although these tags may not be visible in individual tomograms, they may be resolved during sub-volume averaging, revealing how the labelled proteins are organised within the resolved structure. Examples include labelling of proteins with a biotinylation tag using streptavidin, or labelling SNAP-tagged proteins via a benzylguanine-PEG-biotin linker and steptavidin-conjugated 1.4 nm nanogold [51,52]. Both strategies have revealed insights into the structure of *Chlamydomonas* axonemes.

### Future directions

Over the past five years, molecular tagging technologies have advanced significantly, but new developments could further expand their reach and impact. One current challenge is that post-translational targeting creates the possibility for unbound tags, complicating data interpretation. Finding ways to distinguish between bound and unbound tags would greatly improve the utility of these methods. Exogenous tags, such as gold nanoparticles and SPOTs, have been successful in labelling membrane proteins, and expanding their application to intracellular targets is an exciting area for development. Establishing reliable intracellular delivery protocols will be key for this advancement. Additionally, many experiments could benefit from simultaneous labelling of multiple targets, such as investigating the co-localisation of two related proteins. Developing these multiplex labelling strategies would enable molecular tags to address an even wider array of research questions.

#### Distinguishing labelling events from the background

Data analysis is complicated by the presence of unbound tags, particularly in crowded cellular environments where binding cannot be visually confirmed. Lowering the tag concentration reduces the background, but at the cost of labelling efficiency. Combining molecular tagging with cryoCLEM can help. Fluorescent tagging of the target protein enables correlation between FM and cryoET data, confirming the target’s presence in the tag’s vicinity. Another approach could involve coupling the binding of a tag to its target with a conformational change of the tag, providing a visual readout of binding. Nucleic acid nanostructures show promise in this regard, as they can be designed to undergo large-scale conformational changes in response to binding specific DNA sequences or proteins [53,54].

#### Cellular delivery of exogenous tags

For intracellular applications, genetically encoded tags seem the natural choice, but optimising their expression across different cell lines and targets will be labour-intensive. An alternative is to deliver exogenous tags, like SPOTs or gold nanoparticles, directly into cells. Both electrotransfection and microfluidic mechanoporation are proven strategies for delivering both nanoparticles and DNA nanostructures to the cytoplasm [9,55–58].

Technological advancements have improved cell delivery of cargo, reducing cell mortality and improving delivery rates [59]. However, ensuring the diffusion or transport of tags to the correct subcellular location is an additional challenge. This issue also affects genetically encoded non-fusion tags like Ferritags and GEMs. Although progress in trafficking small molecules has been made [60], larger molecules may present greater difficulties.

#### Multiplex labelling

Multiplex labelling could greatly expand the applications of molecular tagging by enabling simultaneous identification of multiple molecules. This capability would enable us to, for example, unravel the exact protein composition of complexes in tomograms. Achieving multiplexing requires multiple distinguishable tags with orthogonal targeting strategies. One approach could use nanoparticles of varying sizes and different targeting moieties, such as nanobodies. In the past, the heterogeneity of gold nanoparticles has limited their scope for multiplexing, but recent advancements in synthesising atomically precise gold nanoparticles have resulted in more uniform structures that are easier to distinguish [61]. Another clear strategy for multiplexing is using nucleic acid nanostructures, which can be designed to adopt a wide range of shapes and sizes and can be conjugated to various binding moieties such as aptamers and nanobodies.

### Outlook

Molecular tags are useful tools that increase the scope and power of cryoET, enabling the identification of specific molecules within the complex cellular environment. Various strategies, including modified gold nanoparticles, Ferritags, SPOTs, and GEMs, have been established to address a range of biological questions. Each method offers unique advantages and presents specific challenges (Table 1), such as balancing contrast with potential imaging artefacts and ensuring precise targeting without disrupting protein localisation. The future developments outlined here will further expand the applicability of molecular tagging.

## Figures and Tables

**Table 1:** Overview of example molecular tag types for cryoET

Molecular tag type	Contrast component	Targeting method	Key advantages	Key limitations
Immunogold labelling	Metal (gold) clusters/nanoparticles	Antibodies/nanobodies	High contrast, well-established method, many sizes and surface chemistries available.	Exogenous tag — requires electroporation for intracellular labelling, strong scattering can cause artefacts in tomograms, non-specific binding, multivalent targeting can cause aggregation of target
Metallothionein	Clusters of metal ions	Direct genetic fusion	Fully cloneable tag, small size	Relatively low contrast, high metal concentrations required, metals can be toxic to cells
Ferritin fusions	Iron core	Direct genetic fusion	Fully cloneable tag, high contrast	High iron concentrations required, multivalent targeting
Ferritag	Iron core (or protein shell)	FRKB/FRB dimerisation	Inducible targeting, expressed in cells, high contrast	High iron concentrations required (for iron-loaded Ferritags), multivalent targeting, large size
SPOTs	Nucleic acids (phosphorous)	Aptamer	Monovalent targeting, asymmetric structure	Exogenous tag currently limited to surface labelling, large size
GEMs	Protein shell (encapsulin)	FKBP/FRB dimerisation + nanobody	Inducible targeting, expressed in cells	Relatively low contrast, multivalent labelling, large size

## Summary

- Electron cryo-tomography is a powerful tool for visualising molecular structures within their native cellular context, providing detailed insights into cellular processes.
- Identifying specific molecules within complex environments is challenging. Molecular tagging aims to overcome this limitation by attaching a high contrast moiety to the molecule of interest, distinguishing it in tomograms.
- Available molecular tagging strategies include modified gold nanoparticles, Ferritags, SPOTs and GEMs.
- Future advances in tagging strategies include improving intracellular delivery of exogenous tags, distinguishing bound tags from the background through a fluorescent or visual readout, and developing multiplex labelling strategies. These developments promise to expand the scope of cryoET imaging.

## Abbreviations

CLEM: correlative light electron microscopy
cryoET: cryogenic electron tomography
EM: electron microscopy
FM: fluorescence microscopy
GEM: genetically encoded multimeric particle
PEG: polyethylene glycol
SNR: signal-to-noise ratio
